# Macronutrient Intake and Insulin Resistance in 5665 Randomly Selected, Non-Diabetic U.S. Adults

**DOI:** 10.3390/nu14050918

**Published:** 2022-02-22

**Authors:** Larry A. Tucker

**Affiliations:** College of Life Sciences, Brigham Young University, Provo, UT 84602, USA; tucker@byu.edu; Tel.: +1-801-422-4927

**Keywords:** carbohydrate, protein, fat, unsaturated fat, saturated fat, sugar, starch, fiber, diabetes

## Abstract

The main goal of this investigation was to evaluate the relationships between several macronutrients and insulin resistance in 5665 non-diabetic U.S. adults. A secondary objective was to determine the extent to which the associations were influenced by multiple potential confounding variables. A cross-sectional design and 8 years of data from the 2011–2018 National Health and Nutrition Examination Survey (NHANES) were used to answer the research questions. Ten macronutrients were evaluated: total carbohydrate, starch, simple carbohydrate, dietary fiber, total protein, total fat, saturated, polyunsaturated, monounsaturated, and total unsaturated fat. The homeostatic model assessment (HOMA), based on fasting glucose and fasting insulin levels, was used to index insulin resistance. Age, sex, race, year of assessment, physical activity, cigarette smoking, alcohol use, and waist circumference were used as covariates. The relationships between total carbohydrate intake (F = 6.7, *p* = 0.0121), simple carbohydrate (F = 4.7, *p* = 0.0344) and HOMA-IR were linear and direct. The associations between fiber intake (F = 9.1, *p* = 0.0037), total protein (F = 4.4, *p* = 0.0393), total fat (F = 5.5, *p* = 0.0225), monounsaturated fat (F = 5.5, *p* = 0.0224), and total unsaturated fat (F = 6.5, *p* = 0.0132) were linear and inversely related to HOMA-IR, with 62 degrees of freedom. Starch, polyunsaturated fat, and saturated fat intakes were not related to HOMA-IR. In conclusion, in this nationally representative sample, several macronutrients were significant predictors of insulin resistance in U.S. adults.

## 1. Introduction

Insulin resistance is a pathological condition in which body cells manifest reduced sensitivity to insulin. Insulin promotes the distribution of glucose across muscle and fat tissues and decreases the liver’s release of glucose by decreasing the breakdown of glycogen, and gluconeogenesis. Additionally, insulin represses the release of non-esterified fatty acids from fat tissue by decreasing lipolysis [1]. Because of insulin resistance, glucose is not transported into cells at the correct rate, resulting in elevated blood glucose levels. As blood glucose levels increase, the body responds by increasing circulating insulin levels. Consequently, individuals who are insulin resistant often have elevated insulin levels.

Some of the most common chronic diseases in developed societies are linked to insulin resistance. For example, the relationship between insulin resistance and the risk of developing cardiovascular disease is strong [2,3,4]. Endothelial dysfunction and the development of atherosclerosis is also closely tied to insulin resistance [5,6], along with stroke [7], hypertension [8], dyslipidemia [9], neurodegenerative diseases [10,11], metabolic syndrome [12], and type 2 diabetes [13], to name a few.

Although there are many factors that contribute to insulin resistance, obesity is one of the strongest driving factors [14], even in children [15]. Abdominal obesity seems to pose a greater risk of metabolic disease than an elevated body mass index [16]. Although challenging, weight loss increases insulin sensitivity and reduces the risk of developing insulin resistance and diabetes.

Regular physical activity also plays a significant role in reducing the likelihood of insulin resistance [17], even in those with abdominal obesity [18]. Furthermore, exercise and physical activity also reduce the risk of developing type 2 diabetes [19].

Although obesity and physical inactivity often lead to metabolic impairment, several other lifestyle factors also contribute to metabolic health. For example, research indicates that diet is a key modifiable variable that can be targeted to counter the rising rates of insulin resistance and diabetes. Research indicates that diet composition can be manipulated to improve insulin sensitivity and reduce the risk of diabetes.

To date, dozens of investigations have been conducted to evaluate the relationship between diet composition and metabolic disease, particularly insulin resistance. Study designs and methods have varied substantially, and findings have been mixed. Due to the inconsistent findings in the literature, additional investigations are needed to assess the relationship between diet composition and insulin resistance. Moreover, most investigations in this area have been conducted using small samples and have resulted in few generalizable findings. Hence, the chief goal of the present study was to determine the extent to which differences in diet composition, particularly macronutrient intake, account for differences in insulin resistance in a large sample of adults representing the U.S. population. Another objective was to ascertain the role of several potential confounding factors, including age, sex, race, year of assessment, smoking, alcohol, physical activity, and waist circumference, on the relationship between macronutrient intake and insulin resistance. Effect modification was also evaluated across tertiles of the primary macronutrients and levels of physical activity. 

## 2. Materials and Methods

### 2.1. Study Design and Sample

The U.S. National Health and Nutrition Examination Survey (NHANES) database was used to answer the research questions. NHANES is an ongoing government-run survey, administered by the National Center for Health Statistics (NCHS) and the Centers for Disease Control and Prevention (CDC). NHANES data were gathered through the use of extensive interviews, questionnaires, blood samples, and physical examinations performed by trained professionals on individuals selected randomly from the U.S. population.

Before data were collected, each individual in the sample provided written informed consent. The Ethics Review Board for the NCHS approved the data collection protocol and the data files, containing no confidential information, that were published on the NHANES website for public use [20].

This investigation used NHANES data gathered during an 8-year period, from 2011–2018. Data from 2019–2020 were not available due to the COVID pandemic limiting the NHANES data collection process. The codes signifying ethical approval for NHANES data collected from 2011–2018 are: Protocol #2011–17 and Protocol #2018-01.

A total of 5665 adults were included in this study, ages 18–75 years. A 4-stage strategy was employed to randomly select non-institutionalized, civilian U.S. adults. Census information was utilized so that counties, then blocks, then dwelling units, and finally individuals were randomly selected, so that the final data are nationally representative [21].

Individuals who reported that they were diabetic, or took oral medication or insulin to control their blood sugar levels, or were found to have a fasting blood glucose concentration of 126 mg/dL or higher, were excluded from the sample. Those with hypoglycemia (fasting glucose < 70 mg/dL) were also excluded. Additionally, subjects who did not consume any kilocalories (kcal) during either of the two 24-h dietary recall assessments (i.e., they fasted) were excluded. Participants with extreme HOMA-IR levels (≥4 standard deviations above the mean) were excluded, and individuals who were underweight (BMI < 18.5) were excluded because of the likelihood of an eating disorder, severe frailty, or a serious disease.

### 2.2. Instrumentation and Measurement Methods

Macronutrient intake was the exposure variable for this study. A total of 10 macronutrients were studied. The outcome variable was insulin resistance, indexed using the homeostatic model assessment for insulin resistance (HOMA-IR). Age, sex, race, year of assessment, physical activity, cigarette smoking, alcohol use, and waist circumference were included as covariates, so their influence on the association between the exposure and outcome variables could be minimized.

#### 2.2.1. Insulin Resistance

HOMA-IR is the most frequently used method of assessing insulin resistance. Over 18,600 published journal articles available in PUBMED include the term “HOMA” or “HOMA-IR.” The HOMA-IR calculation was based on fasting plasma glucose and fasting insulin levels., specifically: fasting insulin (μU/mL) × fasting glucose (mg/dL) ÷ 405. Subjects who were randomly assigned to attend a morning data collection session were asked to fast for 9 h for the fasting blood draw. Comprehensive information is provided by NHANES on their website about the glucose and insulin measurement protocols [22,23].

#### 2.2.2. Macronutrient Intake

Two 24-h dietary recall assessments were used to gather the macronutrient data. The first diet interview occurred in-person. Data for the second recall interview was collected by telephone 3 to 10 days later. The average of the two assessments was used. Both dietary assessments gathered detailed information about all foods and beverages eaten during the 24-h period prior to the interview (midnight to midnight). Individuals reporting that they did not eat during one or both of the 24-h dietary assessment periods were not included in the analyses.

According to Willett, one 24-h dietary recall may be adequate if sample sizes are sufficiently large. He also states that, to estimate within-person variability, “it is statistically most efficient to increase the number of individuals in the sample, rather than to increase the number of days beyond 2 days per individual” (page 55) [24]. Given that the present study included over 5000 randomly selected adults, with each completing two 24-h dietary recalls 3–10 days apart, the assessment methods employed were more than satisfactory to secure quality estimates of dietary intake.

The diet recall interviewers were thoroughly trained in preparation for administering the diet assessments. They each had at least 10 college credits in nutrition courses, and each was a graduate in Food and Nutrition or Home Economics. Each of the individuals administering the diet recalls was bilingual and the dietary data collection occurred in a private setting in the NHANES mobile examination center (MEC). Interviewers were guided by scripts, and the computer-based program afforded a standard interview protocol. The diet assessments followed a multi-pass format called the Automated Multiple Pass Method (AMPM), available online [25]. The diet recall included food probes that have been used in previous United States Department of Agriculture (USDA) and NHANES surveys.

To help the participant, the in-person interviews included a number of real-life examples, such as different sized glasses, bowls, mugs, bottles, spoons, cups, plates, etc. After completing the first (in-person) diet interview, subjects were given sample cups, spoons, etc. and a food model booklet to take home to assist them during the telephone diet interview.

The present investigation included 10 macronutrients in order to study their relationship with insulin resistance: total carbohydrate, starch, simple carbohydrate, and fiber; total protein; total fat, polyunsaturated fat, monounsaturated fat, saturated fat, and total unsaturated fat. Except for dietary fiber intake, each of the macronutrients was reported as a percentage of the total energy consumed by the participant. Hence, total energy intake was taken into account for each macronutrient and for each participant. For fiber intake, the value was expressed as grams per 1000 kcal.

### 2.3. Covariates

NHANES classifies race into six categories: Mexican American, Non-Hispanic Black, Non-Hispanic White, Non-Hispanic Asian, Other Hispanic, or Other Race/Multi-racial. The NHANES racial categories were used as a covariate in the present study.

Waist circumference was utilized to index abdominal or central adiposity. Abdominal adiposity tends to be a better predictor of insulin resistance and diabetes than general obesity [16]. The measurement was taken by trained specialists. The procedures used by those performing the assessment were evaluated regularly to ensure high-quality performance. Measurement of the waist was taken in a customized room in the mobile examination center (MEC). Those taking the measurements were assisted by a trained recorder. The person taking the measurement and the assistant worked together to position, assess, and record the values precisely. The measuring tape was put around the body in a horizontal plane immediately above the top border of the ilium. To safeguard horizontal alignment of the tape, a wall mirror was utilized. The tape was placed snugly around the person, but the skin was not to be compressed. The measurement was finalized at the conclusion of a normal expiration [26].

Differences in physical activity levels were also used as a covariate. Physical activity was measured via interview. Participants were queried about the amount of time they spent in moderate and vigorous activities. Moderate activity was described as physical activity that results in small increases in breathing or heart rate, such as walking, carrying light loads or casual bike riding. Vigorous physical activity was explained as activity resulting in large increases in breathing or heart rate, such as jogging or running, walking up a moderate or steep incline, or lifting heavy loads.

Physical activity (PA) was assessed using specific questions asked by the NHANES interviewer: “In a typical week, on how many days do you do moderate-intensity sports, fitness, or recreational activities?” Also, “How much time do you spend doing moderate-intensity sports, fitness, or recreational activities on a typical day?” These questions, with slight alterations, were also used to assess vigorous PA. For the two intensities, days and minutes were multiplied together to produce the total minutes of moderate and total minutes of vigorous physical activity. These minutes were summed together to give total time (minutes) spent doing moderate and vigorous physical activity (MVPA).

The relationship between each of the primary macronutrients and insulin resistance was evaluated within three different sex-specific categories of physical activity. Participants who reported 0–30 min of activity per week were placed into the Low physical activity category. This category comprised approximately 45% of the sample. The remaining participants were divided equally between the Moderate and High physical activity categories. Specifically, females who reported 40 or more minutes and less than 180 min of activity per week were placed in the Moderate physical activity category. Males who reported 40 or more minutes and less than 240 min of activity per week were also placed in the Moderate physical activity category. Women reporting 180 min or more and men reporting 240 min or more of activity were placed in the High physical activity category.

Statistical adjustments were also made for differences in alcohol use in the current study. As part of the two 24-h dietary recall interviews, participants were asked to report their alcoholic beverage consumption. Those who reported that they did not drink any beverages containing alcohol were given a zero and alcohol consumers were assigned values based on the percentage of their total energy intake derived from alcohol.

Differences in cigarette smoking were also controlled statistically in this study. Smoking was measured by assessing the typical number of cigarettes smoked per day during the past month. An NHANES interviewer specifically asked, “During the past 30 days, on the days that you smoked, about how many cigarettes did you smoke?” Non-smokers were given the value of 0, whereas smokers had values up to 95 [27].

### 2.4. Data Analysis

A multi-level sampling technique was employed in this study so that the findings can be generalized to the U.S. adult population. To accomplish this, strata, clusters and individual sample weights were included in each statistical model.

With a sample size of 5665 individuals, a high level of statistical power would be expected in each statistical model. However, given the sampling strategy employed, degrees of freedom (df) were based on the number of clusters (121) minus the number of strata (58), resulting in 62 df, instead of 5665 df in the denominator.

There was one outcome variable (HOMA-IR) and 10 exposure variables (macronutrient intake) evaluated separately using multiple regression. A number of covariates were controlled statistically to reduce their influence on the relationship between the macronutrients and insulin resistance, specifically age, sex, race, year of assessment, physical activity, smoking, alcohol use, and waist circumference. Macronutrients could not be employed as covariates to determine their influence on the key relationships because of multicollinearity, tested by using the SAS variance inflation factor option (VIF). The VIF was 3.8 for total carbohydrate intake and 3.5 for total fat intake when in the same model. A VIF ≥ 2.5 is considered problematic [28].

Two statistical analysis strategies were employed to measure the associations between the macronutrients and HOMA-IR. First, linear relationships were tested by treating both the exposure and outcome measures as continuous variables and using multiple regression and the SAS SurveyReg procedure. Second, one-way analysis of variance (ANOVA) was employed using the SAS SurveyReg procedure to measure mean differences in HOMA-IR across each macronutrient divided into tertiles. Tertile cut-points were: Total Carbohydrate: <44.10, 44.11–51.85, >51.85; Simple carbohydrate: <16.52, 16.53–23.59, >23.59%; Starch: <24.23, 24.24–29.51, >29.51; Fiber: <6.43, 6.44–9.31, >9.31; Total protein: <13.94, 13.95–17.24, >17.24; Total fat: 31.47, 31.48–37.44, >37.44; Saturated fat: <9.70, 9.71–12.36, >12.36; Unsaturated fat: <20.93, 20.94–25.20, >25.20; Monounsaturated fat: <10.60, 10.61–13.07, >13.07; Polyunsaturated fat: <6.70, 6.71–8.82, >8.82. The macronutrient categories were based on tertiles calculated using percentage of total kilocalories, so energy intake was taken into account for each macronutrient and each participant. Tertiles of total fiber consumption were categorized based on grams consumed per 1000 kilocalories (kcal).

Covariates were controlled using multiple regression and partial correlation, and the Least Squares Means (LSMeans) procedure was used to produce adjusted means. Effect modification was tested by dividing the macronutrients into tertiles and then analyzing the relationships between each primary macronutrient and HOMA-IR within the individual tertiles. Effect modification was also evaluated with physical activity divided into three categories and then analyzing the associations between each primary macronutrient and HOMA-IR within the three categories separately. Physical activity could not be divided into tertiles because over 40% of the subjects reported participating in no regular physical activity each week.

SAS version 9.4 (SAS Institute, Inc., Cary, NC, USA) was used to organized and examine the data. All the statistical tests were two-sided, and alpha was fixed at <0.05 to define statistical significance.

## 3. Results

The sampling strategy used by NHANES included 59 strata and 121 clusters selected randomly. There were 5665 subjects in the sample spread evenly from 2011–2018. Mean age (± SE) was 43.7 ± 0.4 years. Mean (± SE) energy intake for the two independent 24-h recall assessments was 2130 ± 14.0 kilocalories (kcals) per 24 h. Average (± SE) HOMA-IR was 2.76 ± 0.05. Moreover, mean (± SE) intake of the primary macronutrients, expressed as a percentage of total energy intake, was: total carbohydrate (48.0 ± 0.2), total protein (16.1 ± 0.1), and total fat (34.4 ± 0.1). Fiber intake averaged (± SE) 8.5 ± 0.1 g per 1000 kcals. Mean (± SE) waist circumference was 98.4 (±0.4) cm. Table 1 shows the distribution of values across percentiles for each of the key continuous variables. 

Race and sex were treated as categorical variables in this investigation. Results indicated that 66.2% were Non-Hispanic White, 11.0% were Non-Hispanic Black, 8.4% were Mexican American, 6.2% were Other Hispanic, 4.7% were Non-Hispanic Asian, and 3.4% were Other-race or Multi-racial. The sample was comprised of 52.4% women and 47.6% men.

### 3.1. Dietary Carbohydrate and Insulin Resistance

The linear relationship between carbohydrate intake and HOMA-IR was studied with carbohydrate intake and insulin resistance both treated as continuous variables. After adjusting for the covariates (i.e., age, sex, race, year of assessment, smoking, alcohol, physical activity, and waist circumference) total carbohydrate intake was a significant predictor of HOMA-IR with 62 degrees of freedom. Specifically, as percent of kilocalories (kcal) from total carbohydrate increased, HOMA-IR increased linearly (F = 6.7, *p* = 0.0121), as shown in Table 2. Level of simple carbohydrate consumption was also positively and linearly related to HOMA-IR (F = 4.7, *p* = 0.0344). However, percentage of kcals from starch was not predictive of insulin resistance (F = 1.8, *p* = 0.1886). Grams of total fiber intake per 1000 kcals were inversely associated with HOMA-IR (F = 9.1, *p* = 0.0037). As fiber consumption increased, insulin resistance tended to decrease in a linear fashion (Table 2), after adjusting for the covariates.

The association between total carbohydrate consumption and HOMA-IR was also evaluated by comparing mean differences in HOMA-IR across tertile levels of carbohydrate intake (Table 3). As displayed in Table 3, results showed that adults in the lowest tertile of total carbohydrate intake had significantly less insulin resistance compared to the other two tertiles (F = 10.4, *p* = 0.0001). Similarly, those in the lowest tertile of simple carbohydrate consumption had lower HOMA-IR levels than the other two tertiles (F = 4.8, *p* = 0.0117). However, mean HOMA-IR levels did not differ across tertiles of starch intake. Finally, those in the highest one-third of fiber intake had significantly less insulin resistance than the other two tertiles (F = 3.3, *p* = 0.0436), as displayed in Table 3.

### 3.2. Dietary Protein and Insulin Resistance

Percent of kcals derived from dietary protein was also a significant predictor of insulin resistance (F = 4.4, *p* = 0.0393). The association was linear and inverse. As total protein consumption increased, insulin resistance tended to decrease (Table 2). However, when mean HOMA-IR levels were compared across tertiles of total protein intake, mean differences were not significant (Table 3).

### 3.3. Dietary Fat and Insulin Resistance

As displayed in Table 2, total dietary fat intake, recorded as a percentage of total kcals, was a significant predictor of HOMA-IR (F = 5.5, *p* = 0.0225), with 62 df and both measures treated as continuous variables. The relationship was linear and inverse. Specifically, as total fat intake increased, insulin resistance decreased, after controlling for the potential confounding variables. Conversely, the association between polyunsaturated fat consumption and HOMA-IR was not statistically significant (F = 2.6, *p* = 0.1102). However, the relationship between monounsaturated fat and insulin resistance was significant and inverse (F = 5.5, *p* = 0.0224). Additionally, with polyunsaturated and monounsaturated fat intake combined, the association between unsaturated fat intake and HOMA-IR was significant and inverse (F = 6.5, *p* = 0.0132). Level of saturated fat consumption was not predictive of HOMA-IR (F = 2.1, *p* = 0.1513), with the covariates controlled.

With total fat consumption divided into equal categories, mean HOMA-IR levels differed significantly across the tertiles (F = 6.4, *p* = 0.0030), as shown in Table 3. Specifically, subjects in the highest tertile of total fat intake had significantly less insulin resistance compared to those in the middle or lowest tertile of fat consumption. However, mean levels of insulin resistance did not differ significantly across tertiles of saturated, polyunsaturated, or monounsaturated fat intakes (Table 3). However, with poly- and monounsaturated fat intakes combined, mean HOMA-IR levels differed significantly across tertiles of unsaturated fat (F = 4.1, *p* = 0.0218). Specifically, adults in the highest tertile of unsaturated fat intake had significantly lower levels of HOMA-IR compared to the other two tertiles.

### 3.4. Effect Modification

Isolating the association between macronutrient consumption and insulin resistance is challenging. Consuming more of some foods typically results in consuming less of others. Macronutrients are intercorrelated. Using multiple regression and partial correlation to control statistically for differences in a macronutrient, such as dietary fat intake, when studying the relationship between carbohydrate consumption and insulin resistance, typically results in problems associated with multicollinearity, making the findings invalid or uncertain. Although different from partial correlation, testing for effect modification can help clarify the interaction between each macronutrient and insulin resistance.

As displayed in Table 4, when delimited to individual tertiles of total dietary fat intake, carbohydrate consumption was not related to HOMA-IR. Similarly, total protein intake and dietary fat consumption were not associated with insulin resistance within any of the fat intake tertiles (Table 4).

According to Table 5, none of the primary macronutrients were significantly related to insulin resistance within the lowest tertile of protein intake. Similarly, none were associated with insulin resistance when the sample was delimited to the middle tertile of protein consumption. On the other hand, total carbohydrate, protein, and fat intakes were each related to HOMA-IR in adults reporting a high protein diet. The carbohydrate and HOMA-IR association was positive (direct), and the protein and fat intake correlations were negative (inverse).

As revealed in Table 6, among adults reporting a high carbohydrate diet (highest tertile), none of the primary macronutrients were related to HOMA-IR. With the sample delimited to adults in the middle tertile of carbohydrate intake, fat intake was inversely related to HOMA-IR. Among those eating a low carbohydrate diet, protein intake was inversely related and carbohydrate intake was directly associated with insulin resistance. The carbohydrate correlation was borderline significant.

As shown in Table 7, there were no significant relationships between macronutrient consumption and insulin resistance in the Moderate or High physical activity categories. However, among participants who reported Low physical activity, dietary fat intake was inversely related to HOMA-IR and total carbohydrate consumption was associated with HOMA-IR in a direct and significant relationship. Protein intake was not related to insulin resistance within any of the physical activity categories, Low, Moderate, or High.

## 4. Discussion

The chief objective of the present study was to evaluate the relationships between multiple dietary macronutrient intakes and insulin resistance, measured using HOMA-IR, in 5665 randomly selected U.S. adults. Several potential confounding variables were controlled statistically to help isolate the association between the macronutrient intakes and HOMA-IR. Effect modification across tertiles of the primary macronutrients and physical activity were also evaluated.

There were seven major outcomes in this study: (1) Macronutrient intake was predictive of insulin resistance measured by HOMA-IR in U.S. adults. (2) Both total carbohydrate and simple carbohydrate intakes were positively and linearly related to HOMA-IR, but starch consumption was not associated with HOMA-IR. (3) Fiber intake was inversely related to HOMA-IR. (4) Protein intake was inversely associated with HOMA-IR when both variables were treated as continuous, but protein consumption was not associated with HOMA-IR when protein intake was divided into tertiles. (5) Total fat consumption was linearly and inversely related to HOMA-IR, along with monounsaturated fat intake, when both were treated as continuous measures. However, polyunsaturated and saturated fats were not related to HOMA-IR. Unsaturated fat intake (poly- and monounsaturated fats combined) was linearly and inversely related to HOMA-IR, whether or not the association was analyzed with both variables treated as continuous or with unsaturated fat intake divided into tertiles. (6) With dietary fat intake divided into tertiles, none of the primary macronutrients were predictive of HOMA-IR when confined within the tertiles. (7) None of the primary macronutrients were predictive of HOMA-IR within the Moderate or High physical activity categories, but only within the Low physical activity group.

### 4.1. Dietary Carbohydrate

In the present investigation, as carbohydrate consumption increased, insulin resistance increased in a linear fashion. Simple carbohydrate intake followed a similar pattern, but starch intake was not related to insulin resistance. Numerous scientists have studied the relationship between carbohydrate intake and insulin resistance. However, findings focusing on this relationship vary widely [29].

In a cross-sectional analysis of the Framingham Offspring Study by McKeown et al. with 2834 participants, total carbohydrate intake was not associated with HOMA-IR [30], conflicting with the present findings. On the other hand, whole grain intake and fiber consumption were significantly related to lower levels of insulin resistance. When high glycemic index foods were consumed in larger amounts (i.e., typically indicating more simple carbohydrate intake), HOMA-IR was also significantly higher. On the other hand, in a cross-sectional study of 173 south Asian and European men, elevated insulin levels were directly related to carbohydrate intake (*p* = 0.001) [31], similar to this investigation.

Findings from the Inter99 study revealed that grams of sucrose, glucose, and fructose were each inversely related to HOMA-IR, whereas the association was positively associated with daily lactose intake [32]. Each of these relationships was significant or borderline significant. Note that in the Inter99 investigation, simple sugar intakes were expressed as grams per day, not as a percent of total energy intake.

Randomized controlled trials afford a different perspective. For example, Borkman et al. compared the effect of a high carbohydrate diet or a high fat diet (mostly saturated fat) on insulin sensitivity over three weeks [33]. The diets were administered in random order. Whole body glucose uptake using euglycemic glucose clamps revealed no change or difference in the effects of the diets on insulin sensitivity. Similarly, Garg et al. studied eight men with mild diabetes using a randomized cross-over investigation [34]. Diets were isocaloric and either high carbohydrate (60% of kcal) or low carbohydrate (35% of kcal). The low carbohydrate diet was high in monounsaturated fat. Plasma glucose and insulin responses were equal and insulin sensitivity via clamp at the end of each period were not different.

There is evidence that postprandial glycemic and insulinemic responses to foods differ based on the amount and characteristics of the carbohydrate ingested [35,36,37]. As shown in the McKeown investigation above, the glycemic index (GI) can be used to study the degree to which glucose levels are affected by carbohydrate type [36]

According to a randomized cross-over study in diabetic men by Rizkalla et al., four-weeks on a low glycemic diet produced lower postprandial glucose and insulin levels and areas under the curve than 4-weeks on a high GI diet [38]. Overall, whole-body glucose use, assessed using the euglycemic clamp, favored the low glycemic diet [38]. Similarly, Juanola-Falgarona et al. directed a six-month study of 122 overweight or obese adults to evaluate the effect of two moderate-carbohydrate diets or a low-fat diet, each with energy restriction, and different glycemic index scores, on a variety of cardio-metabolic outcomes, including HOMA-IR [39]. Results showed that insulin resistance was decreased more in the low-GI group than the low-fat group.

Several prospective investigations have studied the relationship between the dietary glycemic index and the development of type II diabetes, a common consequence of insulin resistance [40]. Specifically, Villegas et al. studied a cohort of over 64,000 Chinese women with no history of diabetes or other chronic disease for almost five years [41]. Divided into quintiles, the highest GI group had 21% greater risk of developing diabetes than the lowest quintile, and 34% greater risk based on glycemic load. When the focus was simply on carbohydrate intake rather than glycemic index, the highest quintile had 28% higher risk of developing diabetes than the lowest quintile [41]. On the other hand, in a smaller cohort study of Japanese men, Sakurai showed that men in the upper quintile of the glycemic index had 80% higher risk of developing diabetes compared to the lowest quintile, although the fourth and fifth quintiles based on glycemic load did not differ from the lowest quintile [42].

In a five-year prospective study of older Australians by Barclay et al., consumption of total carbohydrate, sugar, starch, and fiber, evaluated separately, were not predictive of the development of type 2 diabetes. However, in adults younger than 70 years old, higher intake levels on the glycemic index were predictive of higher incidence of diabetes over time [43].

Three large prospective cohort investigations, the Nurses’ Health Study [44], the Health Professionals Follow-up Study [45], and the Iowa Women’s Health Study [46] investigated the extent to which total carbohydrate intake influences risk of developing diabetes over time. None of these investigations found an association between total carbohydrate consumption and diabetes incidence. On the other hand, Swishburn et al. designed a five-year prospective cohort investigation which showed that a low fat, moderately high carbohydrate diet was correlated with reduced insulin resistance and decreased risk of developing diabetes in adults with diminished glucose tolerance [47].

### 4.2. Dietary Fiber

Fiber intake and insulin resistance were strongly and inversely related. Similar to the current study, many investigations indicate that diets with high fiber content have beneficial effects on insulin sensitivity. Specifically, Fukagawa et al. studied 12 healthy individuals before and after 3–4 weeks of a high carbohydrate and high fiber diet [48]. Glucose disposal using the euglycemic clamp was measured. The high carbohydrate and high fiber diet reduced fasting glucose and insulin levels substantially and glucose disposal rates were also improved significantly. Similarly, Pereira et al. looked at the effect of a whole grain compared to a refined grain diet on insulin sensitivity using a randomized crossover design in 11 overweight, hyper-insulinemic adults [49]. Energy needs were balanced to prevent weight gain. Insulin sensitivity was significantly better when on the whole grain compared to the refined grain diet.

Using a cross-sectional design and baseline values from the Inter99 study, Lau et al. employed Danish adults to study the association between fiber consumption and HOMA-IR [32]. A food frequency questionnaire (FFQ) was utilized to assess fiber intake. Fiber was reported in total grams, not grams per 1000 kcal. Even after controlling for a variety of covariates, the relationship between fiber intake per day and HOMA-IR remained significant and inverse.

Lutsey et al. studied the association between whole grain intake and HOMA-IR in the MESA (Multi-Ethnic Study of Atherosclerosis) investigation [50]. Findings indicated that as whole grain intake increased, HOMA-IR decreased, even after adjusting for potential mediating factors.

Cross-sectional outcomes were also the focus of the Insulin Resistance Atherosclerosis Study by Liese et al. [51]. Fiber intake was measured by a food frequency questionnaire. Fiber consumption was not reported as grams per 1000 kcal. Findings revealed that fiber intake was associated directly with insulin sensitivity.

In general, it appears that the link between fiber intake and insulin resistance is strong and consistent. Current dietary recommendations in the United States encourage significant amounts of whole-grains and high-fiber foods, consistent with the literature [52].

### 4.3. Dietary Protein

Fewer studies have investigated the relationship between protein intake compared to carbohydrate consumption and insulin resistance. Overall, findings have been mixed. In a large, cross-sectional study that included 5675 non-diabetic subjects, a food frequency questionnaire (FFQ) was employed to measure total protein intake [32]. Across quartiles of insulin resistance, mean protein intake levels, reported as a percentage of total energy, increased as HOMA-IR increased (*p* = 0.001), after adjusting for potential confounders.

Protein consumption has an insulinotropic effect. In short, protein intake results in insulin release, which leads to increased glucose clearance from the blood. Despite these predictable outcomes, insulin resistance findings of short-term intervention trials vary, depending on the characteristics of the sample and the source of the protein. For example, according to Kahleova et al., in a 16-week randomized controlled trial, plant-based protein intake was predictive of reduced insulin resistance, whereas animal protein had the opposite effects [53]. However, most of the protein and insulin resistance associations of Kahleova’s study were no longer significant after controlling for changes in BMI and energy intake, suggesting that nearly all the relationships were driven by weight loss. On the other hand, according to Adevia-Andany et al., the association between animal protein intake and insulin resistance is independent of body mass index [54].

Several other short-term trials show that protein intake has no effect on insulin resistance when the sample is healthy [55,56]. However, not all studies agree. Some indicate that protein consumption reduces plasma insulin levels [57].

In obese subjects, the influence of protein on insulin resistance is unpredictable. As shown in a review by Rietman et al., some investigations indicate that protein intake improves insulin resistance, whereas other studies indicate there is no effect [55]. Still others suggest that the outcome is dependent on whether or not weight is lost [55].

Prospective cohort investigations have also produced varied results. In a cohort study with 1205 subjects, Asghari et al. determined that intake of several individual branch-chain amino acids was related to increased risk of developing insulin resistance over 2.3 years [58]. However, total branch-chain amino acid consumption was not related to incident insulin resistance.

In another prospective cohort investigation, Chen et al. followed 6822 participants for over 20 years [59]. Baseline protein intake, particularly animal protein consumption, was positively related to HOMA-IR, increased risk of pre-diabetes, and diabetes. Total plant protein was not associated with any of the metabolic problems, including insulin resistance or diabetes [59].

According to Rietman et al., increasing amino acid levels in the blood by consuming protein can lead to insulin resistance by preventing muscle glucose transport and phosphorylation of glucose with ensuing decreased glycogen synthesis [55]. Hence, protein intake can contribute to alterations in insulin sensitivity and promote insulin resistance [60]. Long-term investigations are likely to capture this process better than studies of short duration or cross-sectional studies.

### 4.4. Dietary Fat

Research findings also vary concerning the relationship between dietary fat intake and insulin resistance. In a high-quality intervention by Samaha et al., after six months on a high fat diet, insulin sensitivity improved among obese subjects [61]. In another investigation, Bisschop et al. designed a cross-over study using six healthy men [62]. Subjects ate each of three isocaloric diets for 11 days. The diets were low-fat with high carbohydrate, intermediate fat and intermediate carbohydrate, and high fat with low carbohydrate. Insulin sensitivity was measured using the clamp method. The ratio of fat to carbohydrate in the diets had no effect on glucose uptake. However, glucose disposal tended to increase as the fat to carbohydrate ratios in the diets increased.

Bradley et al. used a RCT to test the effect of a low-fat versus a low-carbohydrate weight reduction diet (−500 kcals) on insulin resistance in 24 overweight/obese adults over an 8-week period [63]. Insulin action was measured using the clamp method. Significant weight loss occurred in both groups but there was no difference between the two groups in insulin resistance at the conclusion of the study.

Using a cross-sectional design and 7-day weighed food records, no relationship was found between saturated fat intake and serum insulin concentrations in nearly 200 middle-aged men [31]. However, in a sample of 389 older men, ages 70–89, intake of polyunsaturated fats was inversely associated with insulin levels and saturated fat intake was directly related to insulin concentrations [64]. Additionally, using cross-sectional data from the Inter99 study, total fat intake, expressed as a percentage of total kcal, was not related to quartiles of HOMA-IR [32].

According to Rivellese et al., animal studies indicate that insulin action is not only affected by the amount of fat consumed, but the type of fat also has a significant influence [65]. Specifically, saturated fatty acids tend to increase insulin resistance, whereas long- and short-chain omega-3 fatty acids tend to enhance insulin sensitivity.

As part of the multicenter KANWU study, 162 healthy subjects were fed an isoenergetic diet for 3-months with either a high level of saturated or monounsaturated fatty acids [66]. Insulin sensitivity was 12.5% lower on the saturated fat diet and 8.8% higher when the focus was on monounsaturated fat intake. The difference in insulin sensitivity was not present when total fat intake was high.

In a 16-week RCT by Kahleova and Fleeman et al., overweight subjects were randomized to follow a randomized low-fat vegan (*n* = 38) or control diet (*n* = 37) [67]. HOMA-IR was used to index insulin resistance. The authors concluded that even after adjusting for differences in energy intake and body mass, decreased intakes of saturated and trans fats and increased consumption of polyunsaturated fats decreased fat mass and insulin resistance.

High quality clinical studies designed to assess the effect of dietary fat on insulin resistance typically use isocaloric substitution methods to prevent changes in body weight. This safeguard is crucial for accurately determining the influence of dietary fat on insulin sensitivity. However, one of the most important contributors to insulin resistance is weight gain and obesity. Hence, inhibiting weigh gain by using an isoenergetic diet may result in false judgments about the effect of dietary fat on insulin resistance because weight gain is prevented. In the general public, ad libitum, high fat diets often lead to weight gain [68,69,70]. In short, it is likely that many dietary fat and insulin resistance intervention studies have questionable external validity because of the use of this common substitution strategy.

### 4.5. Effect Modification

Rarely have researchers studied the relationship between diet composition and insulin resistance across tertiles of carbohydrate, protein, and fat intake considered separately. Nor have researchers evaluated the association between diet composition and insulin resistance across categories of physical activity. In the present study, there were several unique effect modification findings. For example, none of the primary macronutrients were related to insulin resistance when evaluated within fat intake tertiles. Specifically, total carbohydrate consumption was not related to insulin resistance in adults when the focus was only on those reporting a low-fat, a moderate-fat, or a high-fat diet. Yet, total carbohydrate consumption was directly associated with insulin resistance when participants were not restricted to fat intake tertiles. These results suggest that the relationship between carbohydrate intake and insulin resistance is partly due to the wide-ranging variation in fat intake across the U.S. diet. In short, when delimited to individual tertiles of fat intake, the shared variation necessary for the carbohydrate and insulin resistance association to manifest itself is not sufficient, but when differences in fat intake are not restricted, carbohydrate intake is a significant predictor of insulin resistance.

The same pattern appears to be true for both protein and fat intakes. Specifically, there was no relationship between either of these primary macronutrients and insulin resistance when the associations were confined to fat intake tertiles. However, like carbohydrate consumption, protein and fat intakes were each significant predictors of insulin resistance when the wide-spread variation in fat intake was not restricted to tertiles. Apparently, differences in total fat intake play an important role in the relationships between macronutrient consumption and insulin resistance in U.S. adults.

Effect modification across tertiles of carbohydrate and protein also seems to affect the macronutrient and insulin resistance associations. This is logical because of the inter-correlations among the macronutrients. However, confining subjects to tertiles of carbohydrate or tertiles of protein appears to have less influence on the macronutrient and HOMA-IR relationships than restricting subjects to tertiles of dietary fat.

Also of interest, each of the three primary macronutrients were related to HOMA-IR, but only within the highest protein intake tertile. Macronutrient consumption was not predictive of insulin resistance in adults reporting low or moderate protein intake.

Effect modification was also tested within three categories of physical activity. Only two of the nine associations were significant and both relationships, carbohydrate and fat, were within the low physical activity category. These findings suggest that macronutrient intake may not play as significant a role in insulin resistance when physical activity levels are moderate or high. It could be that if physical activity levels are sufficient, macronutrient consumption may be less important to the development of insulin resistance.

### 4.6. Application of the Results

As shown in Table 2 and Table 3, total carbohydrate and simple carbohydrate consumption were related directly to insulin resistance in U.S. adults. However, fiber, protein, and dietary fat consumption, particularly unsaturated fat, were inversely related to HOMA-IR. Consequently, the temptation would be to conclude that a diet with less carbohydrate, particularly fewer simple carbohydrates, and with more fiber, protein, and unsaturated fat, should be recommended. Of course, such advice would be overreaching the cross-sectional design of this study. Instead, the present results should be considered as additional evidence supporting other investigations that have found similar dietary patterns associated with insulin resistance.

### 4.7. Intricacies of the Diet and Insulin Resistance Relationship

Dietary relationships are complex. An inherent issue associated with the study of diet composition is that higher consumption of one macronutrient usually translates into lower intake of another. For example, when dietary fat is decreased in the diet, carbohydrate intake is usually increased. Foods are not eaten in isolation. The obvious question is whether the outcome is caused by the increased intake of carbohydrates or the decreased consumption of dietary fat, or some other combination? Randomized controlled trials (RCT) are especially vulnerable to this issue because diets are assigned and manipulated. Compliance is also a concern and can have a significant influence on findings.

Insulin resistance is not a simple phenotype. It appears that different tissues have different levels of sensitivity to insulin. Without question, the complexity surrounding the biology of insulin action has led to multiple interpretations of the relationships between diet composition and insulin resistance [1].

There are a number of investigations in the literature about diet composition and insulin resistance. Comparing these investigations is challenging for a variety of reasons. For example, was the study design cross-sectional, prospective, or based on an intervention? Was it short-term or long-term? Was there a control group? What was the composition of the control diet? Did participants gain weight or lose weight? What was the protein source, animal or plant? What was the composition of the dietary fat, saturated or unsaturated? What was the composition of the carbohydrate, simple or complex? How much fiber was in the diet? Was the fiber soluble or insoluble? Were participants younger or older, normal weight, overweight, or obese? Were subjects diabetic or non-diabetic? Clearly, studies focusing on the relationship between diet composition and insulin resistance have produced mixed results partly because investigations that look similar on the surface often have important differences in their samples, designs, and measurement methods.

### 4.8. Weaknesses and Strengths of the Study

The present investigation had multiple weaknesses. Because the study was based on a cross-sectional design, cause-and-effect conclusions cannot be applied. Additionally, there was only a single measure of protein consumption, i.e., total protein intake. It was not divided by NHANES into animal-derived or plant-based protein. Similarly, fiber intake was not categorized by NHANES as soluble or insoluble, but only as total grams of fiber. Moreover, although two 24-h dietary recalls were obtained from each subject, and the sample was very large (>5000 individuals), in general, the more dietary assessments, the more representative the dietary data will tend to be.

This investigation also had several strengths. First, participants were randomly selected using a four-stage sampling model, making the results generalizable to the U.S. civilian, non-institutionalized adult population. Second, a large (*n* = 5665), multi-racial sample was utilized, including subjects who were Mexican American, Non-Hispanic Black, Non-Hispanic White, Non-Hispanic Asian, Other Hispanic, or Other Race/Multi-racial. Third, carbohydrate intake was divided into total carbohydrate, starch, simple carbohydrate, and fiber. Fat was broken into total fat, polyunsaturated, monounsaturated, unsaturated, and saturated fat. Fourth, a number of potential confounding factors, demographic and lifestyle, were controlled statistically to minimize their influence on the results. Fifth, although the cross-sectional design employed in this study has weaknesses, in correlational research, dietary intake can be studied using an ad libitum perspective because usual macronutrient intake is the focus. The present investigation took advantage of this strength. Participants were required to report what they had eaten during the previous 24 h, on two different days. As a result, degree of insulin resistance was evaluated based on usual macronutrient consumption, without concern for artificial manipulation of intake, or lack of compliance among participants.

## 5. Conclusions

In conclusion, evidence from the present investigation, conducted using participants representing the U.S. adult population, clearly shows that diet composition accounts for differences in insulin resistance. Higher intakes of carbohydrate predicted higher levels of insulin resistance. However, a closer look indicated that starch consumption was not related to insulin resistance, and fiber intake fits hand-in-hand with increased insulin sensitivity. Furthermore, elevated consumption of simple carbohydrates was predictive of higher levels of insulin resistance. Additionally, higher intakes of protein predicted lower levels of insulin resistance, but not with protein intake divided into tertiles. Also, higher amounts of fat consumption, particularly unsaturated fat, predicted lower levels of insulin resistance. Adjusting for differences in many demographic and lifestyle variables seemed to have little influence on the relationships. However, testing for effect modification indicated that the wide-range of dietary fat intake in the U.S. may play an important role in the macronutrient and insulin resistance associations. Moreover, evaluation of effect modification also showed that moderate to high levels of physical activity may reduce the role of macronutrient intake on insulin resistance. Overall, because numerous studies focusing on diet composition and insulin resistance have been conducted to date with broadly differing results, more investigations are needed to untangle the complex associations between diet composition and insulin resistance.

## Figures and Tables

**Table 1:** Percentile values of the key variables for 5665 adults representing the U.S. population.

	Percentile				
Variable	10th	25th	50th	75th	90th
HOMA-IR	0.86	1.30	2.06	3.43	5.37
Energy Intake (kilocalories)	1224	1570	2032	2585	3168
Carbohydrate intake (% of kcal)	36.10	41.94	48.06	54.05	59.93
Starch intake (% of kcal)	19.16	22.81	26.88	31.06	35.57
Sugar intake (% of kcal)	10.29	14.49	19.97	25.89	32.28
Fiber intake (grams per 1000 kcal)	4.37	5.86	7.73	10.34	13.49
Protein intake (% of kcal)	11.21	13.16	15.53	18.38	21.66
Fat intake (% of kcal)	25.32	29.64	34.48	39.11	43.41
Saturated fat intake (% of kcal)	7.18	9.04	10.99	13.19	15.25
Polyunsaturated fat intake (% of kcal)	4.94	6.16	7.76	9.49	11.57
Monounsaturated fat intake (% of kcal)	8.29	9.91	11.79	13.81	15.98
Alcohol intake (% of kcal)	0	0	0	3.26	9.78
Smoking (cigarettes per day)	0	0	0	0	9.37
Physical activity (minutes of MVPA per wk)	0	0	59	238	478
Waist circumference (cm)	78.74	86.65	96.57	107.73	119.00
Age (years)	22.26	30.04	42.73	55.85	65.02

HOMA-IR is the homeostatic model of assessment. % of kcal is the percentage of total kilocalories derived from the listed macronutrient. MVPA is moderate to vigorous physical activity.

**Table 2:** Linear relationship between macronutrient consumption and HOMA-IR in a randomly selected sample of 5665 adults representing the U.S. population.

	HOMA-IR			
Dietary Intake Variable	Regression Coefficient (Slope)	SE	F	*p*
Total Carbohydrate	0.011	0.004	6.7	0.0121
Simple Carbohydrate	0.008	0.004	4.7	0.0344
Starch	0.008	0.006	1.8	0.1886
Fiber	−0.023	0.008	9.1	0.0037
Total Protein	−0.016	0.008	4.4	0.0393
Total Fat	−0.013	0.005	5.5	0.0225
Polyunsaturated Fat	−0.017	0.011	2.6	0.1102
Monounsaturated Fat	−0.030	0.013	5.5	0.0224
Unsaturated Fat	−0.016	0.006	6.5	0.0132
Saturated Fat	−0.018	0.013	2.1	0.1513

Note: Each of the dietary intake variables was reported as the percentage of total kilocalories consumed by the participant. For example, if an individual consumed an average of 2000 kilocalories per day for the two 24-h dietary recall assessments, and the person averaged 250 g of carbohydrate intake per day, then the individual’s total carbohydrate intake would be 50% of total kcals (250 g × 4 kcal = 1000 kcal; 1000/2000 = 0.50). The covariates for each model were age, sex, race, year of assessment, smoking, alcohol, physical activity, and waist circumference. There were 62 degrees of freedom (df) in the denominator for each model.

**Table 3:** Differences in mean HOMA-IR levels across tertiles of macronutrient intake in 5665 U.S. adults, after adjusting for covariates.

	Macronutrient Category (Tertiles)				
	Low	Moderate	High		
Macronutrient	HOMA Mean ± SE	HOMA Mean ± SE	HOMA Mean ± SE	F	*p*
Total Carbohydrate	2.7 ^a^ ± 0.06	3.1 ^b^ ± 0.06	3.0 ^b^ ± 0.07	10.4	0.0001
Simple Carbohydrate	2.8 ^a^ ± 0.06	3.0 ^b^ ± 0.07	3.0 ^b^ ± 0.07	4.8	0.0117
Starch	2.8 ^a^ ± 0.08	2.9 ^a^ ± 0.06	3.0 ^a^ ± 0.06	1.0	0.3759
Fiber	3.0 ^a^ ± 0.08	3.0 ^a^ ± 0.07	2.8 ^b^ ± 0.06	3.3	0.0436
Total Protein	3.0 ^a^ ± 0.08	3.0 ^a^ ± 0.06	2.8 ^a^ ± 0.05	1.5	0.2336
Total Fat	3.0 ^a^ ± 0.07	3.0 ^a^ ± 0.06	2.7 ^b^ ± 0.06	6.4	0.0030
Saturated Fat	3.0 ^a^ ± 0.07	2.9 ^a^ ± 0.06	2.8 ^a^ ± 0.06	1.6	0.2180
Unsaturated Fat	3.0 ^a^ ± 0.06	3.0 ^a^ ± 0.06	2.8 ^b^ ± 0.06	4.1	0.0218
Monounsaturated Fat	3.0 ^a^ ± 0.08	3.0 ^a^ ± 0.07	2.8 ^a^ ± 0.06	2.2	0.1194
Polyunsaturated Fat	3.0 ^a^ ± 0.06	3.0 ^a^ ± 0.06	2.8 ^a^ ± 0.06	1.8	0.1752

^a,b^ Means on the same row with the same superscript letter do not differ significantly. The difference in HOMA-IR between the moderate and high fiber groups was borderline significant (*p* = 0.0915). All means were adjusted for differences in the covariates: age, sex, race, year of assessment, physical activity, smoking, alcohol, and waist circumference. Tertile cut-points were: Total Carbohydrate: <44.10%, 44.10–51.85%, >51.85; Sugar: <16.52%, 16.53–23.59%, >23.59%; Starch: <24.23, 24.24–29.51, >29.51; Fiber: <6.43, 6.44–9.31, >9.31; Total protein: <13.94, 13.95–17.24, >17.24; Total fat: 31.47, 31.48–37.44, >37.44; Saturated fat: <9.70, 9.71–12.36, >12.36; Unsaturated fat: <20.93, 20.94–25.20, >25.20; Monounsaturated fat: <10.60, 10.61–13.07, >13.07; Polyunsaturated fat: <6.70, 6.71–8.82, >8.82. The macronutrient categories were based on tertiles calculated using percentage of total kilocalories. Tertiles of fiber intake were categorized based on grams consumed per 1000 kilocalories.

**Table 4:** Linear relationships between the primary macronutrients and HOMA-IR within tertiles of dietary fat intake, after controlling for the covariates.

	Total Dietary Fat Intake								
	Lowest Tertile Only			Middle Tertile Only			Highest Tertile Only		
Predictor	Regression Coefficient ± SE	F	*p*	Regression Coefficient ± SE	F	*p*	Regression Coefficient ± SE	F	*p*
Carbohydrate	0.001 ± 0.007	0.0	0.9274	0.010 ± 0.012	0.7	0.4056	0.009 ± 0.008	1.5	0.2203
Protein	−0.011 ± 0.010	1.1	0.2901	−0.019 ± 0.014	2.3	0.1390	−0.019 ± 0.014	1.9	0.1725
Fat	−0.010 ± 0.014	0.5	0.4795	−0.012 ± 0.033	0.1	0.7213	−0.006 ± 0.012	0.2	0.6415

SE = standard error of the regression coefficient. Total dietary fat intake was divided into tertiles. The relationships between insulin resistance (HOMA-IR) and consumption of total carbohydrate, total protein, and total fat were evaluated separately within each of the three dietary fat tertiles. Age, sex, race, year of assessment, physical activity, smoking, alcohol, and waist circumference were the covariates. Each of the analyses had 62 degrees of freedom (df) in the denominator.

**Table 5:** Linear relationships between the primary macronutrients and HOMA-IR within tertiles of dietary protein intake, after controlling for the covariates.

	Total Protein Intake								
	Lowest Tertile Only			Middle Tertile Only			Highest Tertile Only		
Predictor	Regression Coefficient ± SE	F	*p*	Regression Coefficient ± SE	F	*p*	Regression Coefficient ± SE	F	*p*
Carbohydrate	0.005 ± 0.008	0.4	0.5384	0.006 ± 0.008	0.5	0.4800	0.021 ± 0.007	10.0	0.0025
Protein	0.025 ± 0.035	0.5	0.4821	−0.077 ± 0.060	1.7	0.1969	−0.028 ± 0.011	6.4	0.0137
Fat	−0.009 ± 0.008	1.5	0.2262	−0.005 ± 0.008	0.5	0.5054	−0.022 ± 0.007	8.7	0.0045

SE = standard error of the regression coefficient. Total dietary protein intake was divided into tertiles. The relationships between insulin resistance (HOMA-IR) and consumption of total carbohydrate, total protein, and total fat were evaluated separately within each of the three dietary protein tertiles. Age, sex, race, year of assessment, physical activity, smoking, alcohol, and waist circumference were the covariates. Each of the analyses had 62 degrees of freedom (df) in the denominator.

**Table 6:** Linear relationships between the primary macronutrients and HOMA-IR within tertiles of dietary carbohydrate intake, after controlling for the covariates.

	Total Carbohydrate Intake								
	Lowest Tertile Only			Middle Tertile Only			Highest Tertile Only		
Predictor	Regression Coefficient ± SE	F	*p*	Regression Coefficient ± SE	F	*p*	Regression Coefficient ± SE	F	*p*
Carbohydrate	0.018 ± 0.009	3.7	0.0585	−0.006 ± 0.030	0.1	0.8310	0.006 ± 0.010	0.4	0.5459
Protein	−0.023 ± 0.011	5.1	0.0281	0.031 ± 0.017	3.2	0.0775	−0.014 ± 0.016	0.8	0.3861
Fat	−0.001 ± 0.009	0.0	0.8874	−0.034 ± 0.017	4.1	0.0476	−0.005 ± 0.010	0.2	0.6419

SE = standard error of the regression coefficient. Total carbohydrate intake was divided into tertiles. The relationships between insulin resistance (HOMA-IR) and consumption of total carbohydrate, total protein, and total fat were evaluated separately within each of the three dietary carbohydrate tertiles. Age, sex, race, year of assessment, physical activity, smoking, alcohol, and waist circumference were the covariates. Each of the analyses had 62 degrees of freedom (df) in the denominator.

**Table 7:** Linear relationships between the primary macronutrients and HOMA-IR within 3 categories of physical activity, after controlling for the covariates.

	Physical Activity Category								
	Low Physical Activity			Moderate Physical Activity			High Physical Activity		
Predictor	Regression Coefficient ± SE	F	*p*	Regression Coefficient ± SE	F	*p*	Regression Coefficient ± SE	F	*p*
Carbohydrate	0.013 ± 0.006	4.0	0.0497	0.009 ± 0.006	2.6	0.1156	0.009 ± 0.007	1.4	0.2466
Protein	−0.009 ± 0.013	0.5	0.5033	−0.018 ± 0.011	2.3	0.1309	−0.015 ± 0.009	2.1	0.1487
Fat	−0.017 ± 0.007	5.7	0.0203	−0.010 ± 0.008	1.3	0.2551	−0.009 ± 0.008	1.2	0.2801

SE = standard error of the regression coefficient. Total physical activity was divided into 3 categories. Adults with Low weekly physical activity (30 min or less per week) comprised 45% of the sample. The remaining 55% was divided into sex-specific equal categories with approximately 27.5% of the sample in each. For females, the cut-point dividing between Moderate and High physical activity was 180 min per week. For males, the cut-point was 240 min per week. The relationships between the primary macronutrients and insulin resistance (HOMA-IR) were evaluated separately within each of the three physical activity categories. Age, sex, race, year of assessment, smoking, alcohol, and waist circumference were the covariates. Each of the analyses had 62 degrees of freedom (df) in the denominator.

## Data Availability

All data supporting reported results can be found online as part of the National Health and Nutrition Examination Survey (NHANES). The data are free and can be found at the following website: https://wwwn.cdc.gov/nchs/nhanes/Default.aspx. (accessed on 21 February 2022).
